# Deciphering the secretome of leukocyte-platelet rich fibrin: towards a better understanding of its wound healing properties

**DOI:** 10.1038/s41598-020-71419-7

**Published:** 2020-09-03

**Authors:** Lidia Hermida-Nogueira, María N. Barrachina, Luis A. Morán, Susana Bravo, Pedro Diz, Ángel García, Juan Blanco

**Affiliations:** 1 Platelet Proteomics Group, Center for Research in Molecular Medicine and Chronic Diseases (CIMUS), and Instituto de Investigación Sanitaria de Santiago de Compostela (IDIS), Universidade de Santiago de Compostela, Avda de Barcelona s/n, 15782 Santiago de Compostela, Spain; 2 Proteomics Unit, Instituto de Investigación Sanitaria de Santiago de Compostela (IDIS), Santiago de Compostela, Spain; 3 Medical-Surgical Dentistry Research Group (OMEQUI), Faculty of Medicine and Odontology, Instituto de Investigación Sanitaria de Santiago de Compostela (IDIS), Universidade de Santiago de Compostela (USC), Santiago de Compostela, Spain; 4 Periodontology Unit, Medical-Surgical Dentistry Research Group (OMEQUI), Faculty of Medicine and Odontology, Instituto de Investigación Sanitaria de Santiago de Compostela (IDIS), Universidade de Santiago de Compostela (USC), Santiago de Compostela, Spain

**Keywords:** Proteomics, Protein-protein interaction networks

## Abstract

Leukocyte-platelet rich fibrin (L-PRF) is extensively used in the dentistry field and other clinical scenarios due to its regeneration properties. The goal of the present study was to depict the L-PRF secretome and how it changes over time. We obtained L-PRF membranes and cultured them in DMEM. The secretome was collected at days 3, 7 and 21. The secretome at day 3 was analysed by LC–MS/MS and differences over time were analysed by Sequential Window Acquisition of all Theoretical Mass Spectra (SWATH). Overall, 705 proteins were identified in the secretome of L-PRF membranes after 3 days of culture, including growth factors (EGF, PDGFA) and proteins related to platelet and neutrophil degranulation. A total of 202 differentially secreted proteins were quantified by SWATH when comparing secretomes at days 3, 7 and 21. Most of them were enriched at day 3 such as MMP9, TSP1 and CO3. On the contrary, fibrinogen and CATS were found down-regulated at day 3. Growth factor and western blotting analysis corroborated the proteomic results. This is the most detailed proteome analysis of the L-PRF secretome to date. Proteins and growth factors identified, and their kinetics, provide novel information to further understand the wound healing properties of L-PRF.

## Introduction

In the last two decades, the use of platelet-rich concentrates (PRC) has become very popular in different fields, especially in dentistry, due to their regeneration properties. Different scientists and companies have developed methods to obtained PRC with presence or absence of leukocytes but all of them with addition of anticoagulants to the tubes. In 2001, Choukroun et al. developed the second generation of PRC, Leukocyte platelet rich fibrin (L-PRF)^1^, which is obtained by blood centrifugation without anticoagulant in the tubes. Nowadays, L-PRF is the most commonly used PRC since it is easy to obtain and can be applied as a gel or compressed into a membrane depending on the application site. It has been mostly used in a dentistry field, especially for the treatment of osteonecrosis^2,3^, implant placement in bone-deficient sites^4^ and sinus floor elevation^5^. Additionally, L-PRF has started to be used in other clinical scenarios, for example for the treatment of chronic wounds, leg ulcers or diabetic foot^6^ or for the treatment of ocular lesions^7^.

Wound healing is a complex process, which encompass different phases: haemostasis, inflammation, proliferation and remodelling. In each phase, different cell types take part in order to regenerate the tissue. Two types of blood cells, platelets and leukocytes, are among the cells that participate first. Growth factors, cytokines and other proteins released by these blood cells have chemoattractant properties that contribute to wound repair^8–10^.

In recent years, many studies have analysed in vitro the impact of PRC in order to evaluate proliferation, migration, and differentiation of different cell types^11–14^. At the same time, other groups have tried to figure out which proteins provide the regeneration properties to PRC. Nevertheless, some of them have only measured the concentration of specific growth factors^15,16^, have compared growth factor release in different PRC^17^, or have measured their kinetics^18,19^. In fact, few studies have tried to analyse the PRC secretome in detail^17,20,21^. Differences in the study design and the types of samples analysed did not allow knowing the protein composition of the PRC releasate, specifically in the case of L-PRF. For this reason, it is important to obtain an overall picture of L-PRF releasate protein composition in order to better understand the impact of the L-PRF in wound healing. In the present study, our aim was to identify the proteins released by L-PRF membranes cultured in vitro and differences depending on the incubation time, which could reassemble what happens during the time of treatment with L-PRF in vivo.

## Results

### Secretome profile of L-PRF membranes at day 3 of culture

L-PRF membranes were obtained from blood samples. Due to their blood origin, membranes were washed twice in the first 24 h in order to eliminate the majority of plasma proteins, which could interfere with the identifications of less abundant proteins present in the secretome.

In order to elucidate the secretome profile of L-PRF membranes at day 3 of culture, secretome samples were collected at day 3 from four donors, pooled and analysed following two complementary gel-based proteomic approaches. The aim of carrying out two complementary methodologies was to make more comprehensive the analysis. The first approach was based on concentrating the proteins by SDS-PAGE in one band and excised it. A second approach consisted in running a complete SDS-PAGE electrophoresis and cut the proteome profile into multiple bands. Finally, protein bands were in-gel digested with trypsin and analysed by LC–MS/MS.

Overall, 705 proteins were identified. Both approaches presented a certain degree of overlap (235 proteins), although many proteins were exclusively identified by one of the methodologies. Indeed, concentrating proteins in a band showed 169 unique proteins, among them growth factors such as TGFB1 and Latent-transforming growth factor beta-binding protein 1 (LTBP1). Growth factors were also present among the 301 unique proteins identified following protein separation by SDS-PAGE, such as PDGFA, EGF and HDGF.

Some examples of proteins identified by both approaches include Fibronectin (FINC) and some Fibrinogen chains (FIBG, FIBA), related to coagulation system and acute phase response; proteins linked to clathrin, such as AP2- complex subunit alpha-1 (AP2A1), and Clathrin interactor 1 (EPN4); Integrin beta-1 (ITB1); Ras-related protein (RAB7A); and Platelet glycoprotein 4 (CD36), implicated in LXR/RXR activation. The complete list of identifications by both approaches is shown in Supplementary Table 1.

**Table 1:** Growth factors quantified in L-PRF secretomes at days 3 and 7.

Growth factors analysed				
AREG	**BDNF**	**FGF2**	BMP4	BMP5
**BMP7**	**NGF**	**EGF**	EGFR	PROK1
FGF4	**FGF7**	*GDF15*	GDNF	**SOMA**
HBEGF	**HGF**	**IBP1**	**IBP2**	IBP3
**IBP4**	**IBP6**	IGF1	**INS**	**CSF1R**
TNR16	**NTF3**	NTF4	**TR11B**	**PDGFA**
PLGF	KITLG	**KIT**	TGFA	TGFB1
**TGFB3**	VEGFA	**KDR**	**FLT4**	FIGF

Bold indicates higher concentration at day 3; italics indicates higher concentration at day 7.

**Figure 1:**
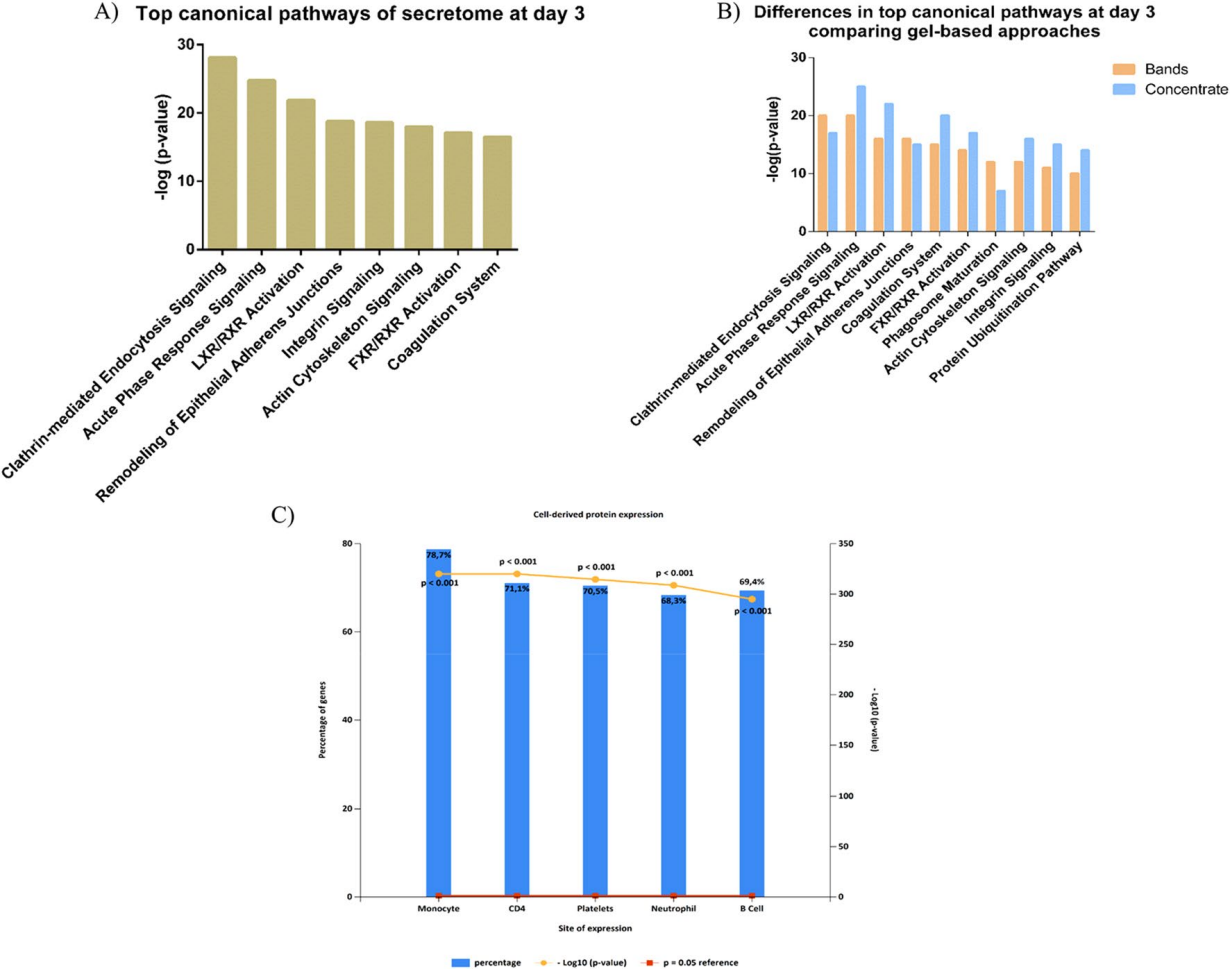
Systems biology analysis of the L-PRF secretome. **(A)** Representation of relevant canonical pathways in which the identified proteins at day 3 are involved. **(B)** Representation of principal canonical pathways related to proteins identified at day 3 comparing the two gel-based approaches employed. C) Cell-derived expression of proteins identified at day 3.

The systems biology analysis showed that top canonical pathways from the total number of identifications were clathrin-mediated endocytosis, acute phase response signalling and LXR/RXR activation, among others (Fig. 1A). Moreover, these pathways were found in the analysis of data for each of the approaches but changing positions in the list, basically because of the bigger number of identifications obtained by separating proteins by SDS-PAGE and the different proteins found between methodologies (Fig. 1B).

A complementary String data analysis showed regulated exocytosis, vesicle-mediated transport and secretion as principal biological pathways related to the proteins identified. In addition, the principal cellular component of proteins identified at day 3 was secretory vesicles and other secretory variants. The presence of proteins related to platelet extracellular vesicles (CD9, Integrin alpha-IIb (ITA2B)) and neutrophil-derived microparticles (Azurocidin (CAP7), Myeloperoxidase (PERM), Bactericidal permeability-increasing protein (BPI), Cathepsin G (CATG), Matrix metalloproteinase-9 (MMP9)) strongly indicate the presence of vesicle release. However, this does not mean that the proteins identified are only present in platelet and neutrophil-derived extracellular vesicles; FunRich reveals that proteins identified at day 3 also derived from monocyte, CD4 lymphocytes and B cells (Fig. 1C).

### Differential 1D-SDS-PAGE profile analysis between secretomes at days 3 and 7

In order to identify differences in the L-PRF secretome at days 3 and 7, a 1D-SDS-PAGE analysis was performed. Protein samples (from the secretomes collected at days 3 and 7) from four donors were pooled and loaded on an 11% bis–tris acrylamide gel. Following gel staining, four main bands were clearly different in intensity between conditions (Supplementary Fig. 1). Bands were sliced, digested with trypsin and analysed by LC–MS/MS.

A total of 371 proteins were found at day 3, and 292 at day 7, and 259 were identified in both conditions. Common identifications belong to secretory pathways; indeed, proteins such as CD9, ITA2B and CAP7, CATG are related to extracellular vesicles, platelet and neutrophil-derived, respectively.

Focusing on identifications only found in a unique condition, growth factors such as EGF and EGF-containing fibulin-like extracellular matrix protein 1 (FBLN3) were identified at day 3. On the contrary, leukocyte adhesion proteins (Intercellular adhesion molecule 3 (ICAM3) and Myosin light polypeptide 6 (MYL6)) were only found at day 7 condition. The complete list of identifications present in the differential bands analysed at both days is shown in Supplementary Table 1.

### Growth factor quantitative analysis complements and corroborates the qualitative proteomic data

Given the relevance of the presence of growth factors in the secretome, an ELISA growth factor analysis was performed complementing the proteomic approach.

Secretomes collected at days 3 and 7 were used for array hybridization at a concentration of 500 µg/mL, following the manufacturer´s protocol. A total of 40 growth factors from different families and with different function were quantified (Table 1).

**Figure 2:**
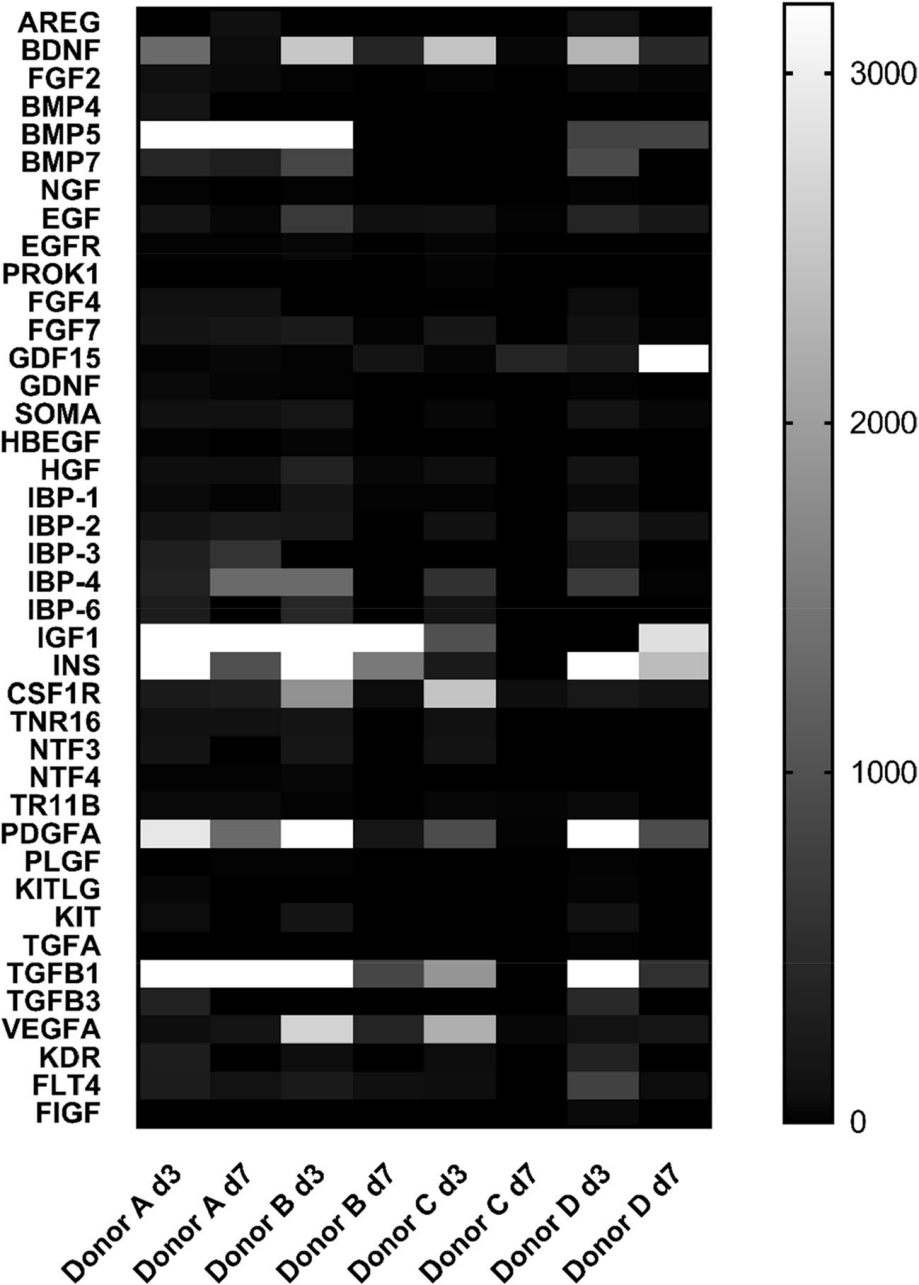
Growth factor analysis. Heatmap shows differential expression of 40 growth factors in L-PRF secretome between four donors **(A–D)** at day 3 (d3) and day 7 (d7). The color code indicates concentrations of growth factors expressed in pg/ml, ranging from black (low concentration) to white (high concentration). The figure was created using GraphPad Prism version 7.00 for Windows, GraphPad Software, La Jolla CA USA, https://www.graphpad.com.

Due to the high variability observed (Fig. 2) only growth factors found in one condition in at least 3/4 donors were considered for the analysis. Following this criteria, 21 growth factors were found at higher concentrations at day 3 versus day 7 (BDNF, FGF2, EGF, SOMA, HGF, IBP1, INS, TR11B, PDGFA, KDR, FLT4, BMP7, NGF, FGF7, IBP2, IBP4, IBP6, CSF1R, NTF3, KIT, TGFB1). Only one growth factor was found increased at day 7 versus day 3 in all donors, growth differentiation factor 15 (GDF15).

As expected, some growth factors analysed in the array were previously identified by LC–MS/MS in the secretome profile analysis at day 3, for example EGF, PDGFA and TGFB1. Actually, these growth factors previously found in the proteomic analysis were found among the highest concentration in the array analysis, showing a correlation between techniques.

### SWATH analysis: protein enrichment at day 3 of culture

In order to further complement the initial differential proteomic profile analysis shown above, an independent cohort of donor samples were collected to analyse quantitative differences in the L-PRF secretome at days 3, 7 and 21 of culture by SWATH analysis.

To do so, protein samples from four different donors were pooled on equal amounts at different conditions. An equal amount of protein of each pool sample was put together in order to generate a library. Afterwards, samples were quantified in triplicate following the SWATH acquisition method.

In total, we found 202 proteins differential regulated between conditions (p-value < 0.05). More precisely, 187 of these proteins were found up-regulated at day 3 and their amount diminished over time. This is the case of proteins related to platelet degranulation and activation such as Thrombospondin-1 (TSP1), TGFB1, complement precursors (CO3, CO4A and COA4B), CD9 and P-selectin (LYAM3) (Table 2). Other examples were found in proteins related to neutrophil degranulation, for example some histones, MMP9, Lactotransferrin (TRFL), PERM, CAP7, Neutrophil defensin 3 (DEF3); and haemostasis process, such as platelet glycoproteins (e.g. GP1BB and GPV). Nevertheless, not all proteins identified by SWATH were enriched at day 3. Four proteins, Carbonic anhydrase 1 (CAH1), FIBA, Peroxiredoxin-2 (PRDX2) and Cathepsin D (CATD) showed increased levels over time in all conditions analysed (Table 2). Interestingly, there were also proteins identified that increased their amount over time in 2 out of 3 conditions analysed, for example fibrinogen beta and gamma isoforms (FIBB and FIBG); another type of cathepsin, Cathepsin S (CATS); and some haemoglobin isoforms (HBD, HBB and HBA), among others. The complete list of identified proteins by SWATH is shown in Supplementary Table 2.

**Table 2:** Selection of proteins found differentially regulated in L-PRF secretomes comparing days 3, 7 and 21 (d3, d7, d21) by SWATH (p-value < 0.05).

Uniprot identifier	Protein name	Fold change d3 vs d7	Fold change d3 vs d21	Fold change d7 vs d21
TSP1_HUMAN	Thrombospondin-1 (Glycoprotein G)	4.51	19.78	4.39
TGFB1_HUMAN	Transforming growth factor beta-1 proprotein	4.10	12.97	3.16
CO3_HUMAN	Complement C3	2.96	5.41	1.82
CO4A_HUMAN	Complement C4-A	1.96	1.91	1.58
CO4B_HUMAN	Complement C4-B	1.84	2.91	
CD9_HUMAN	CD9 antigen	4.56	85.95	18.83
LYAM3_HUMAN	P-selectin	5.50	34.74	6.31
MMP9_HUMAN	Matrix metalloproteinase-9	2.00	3.80	1.91
TRFL_HUMAN	Lactotransferrin	3.20	11.50	3.59
PERM_HUMAN	Myeloperoxidase	3.27	50.40	15.41
CAP7_HUMAN	Azurocidin	4.02	18.64	4.64
DEF3_HUMAN	Neutrophil defensin 3	2.34	4.48	1.91
GP1BB_HUMAN	Platelet glycoprotein Ib beta chain	1.64	3.22	1.97
GPV_HUMAN	Platelet glycoprotein V	6.69	62.86	9.39
CAH1_HUMAN	Carbonic anhydrase	0.30	0.16	0.54
FIBA_HUMAN	Fibrinogen alpha chain	0.63	0.50	0.80
PRDX2_HUMAN	Peroxiredoxin-2	0.43	0.22	0.52
CATD_HUMAN	Cathepsin D	0.05	0.01	0.16
FIBB_HUMAN	Fibrinogen beta chain	0.59	0.55	
FIBG_HUMAN	Fibrinogen gamma chain	0.63	0.56	
CATS_HUMAN	Cathepsin S	0.57	0.51	
HBD_HUMAN	Hemoglobin subunit delta	0.43		1.76
HBB_HUMAN	Hemoglobin subunit beta	0.43		2.04
HBA_HUMAN	Hemoglobin subunit alpha	0.46		2.69

A fold change above 1 between conditions A vs B indicates that the variation is favourable to condition A.

Many of the proteins quantified by SWATH were also identified in both conditions (days 3 and 7) in the differential 1D-SDS-PAGE analysis (TSP1, CO3, CO4A, CD9, MMP9, CAP7, FIBA, PRDX2, CATS). However, GPV and CAH1, were only identified at day 3 and day 7, respectively in the 1D-SDS-PAGE analysis.

### MMP9, TSP1 and CO3 are up-regulated and fibrinogen and CATS are down-regulated at day 3 of culture

Some of the proteins identified in the SWATH differential analysis were validated by western blot in an independent cohort of secretome samples. We selected three proteins that decreased (MMP9, TSP1 and CO3), and two that increased their levels over time (Fibrinogen and CATS) for these validation studies. These proteins were selected because they were also previously found in the differential 1D-SDS-PAGE-based analysis. In addition, MMP9, TSP1 and CO3 are related with neutrophil and platelet degranulation, the principal biological processes that are occurring in the L-PRF membranes. Fibrinogen and CATS are also associated with platelet and neutrophil degranulation, respectively. Moreover, an increase in the level of fibrinogen and CATS over time could indicate cell apoptosis processes.

**Figure 3:**
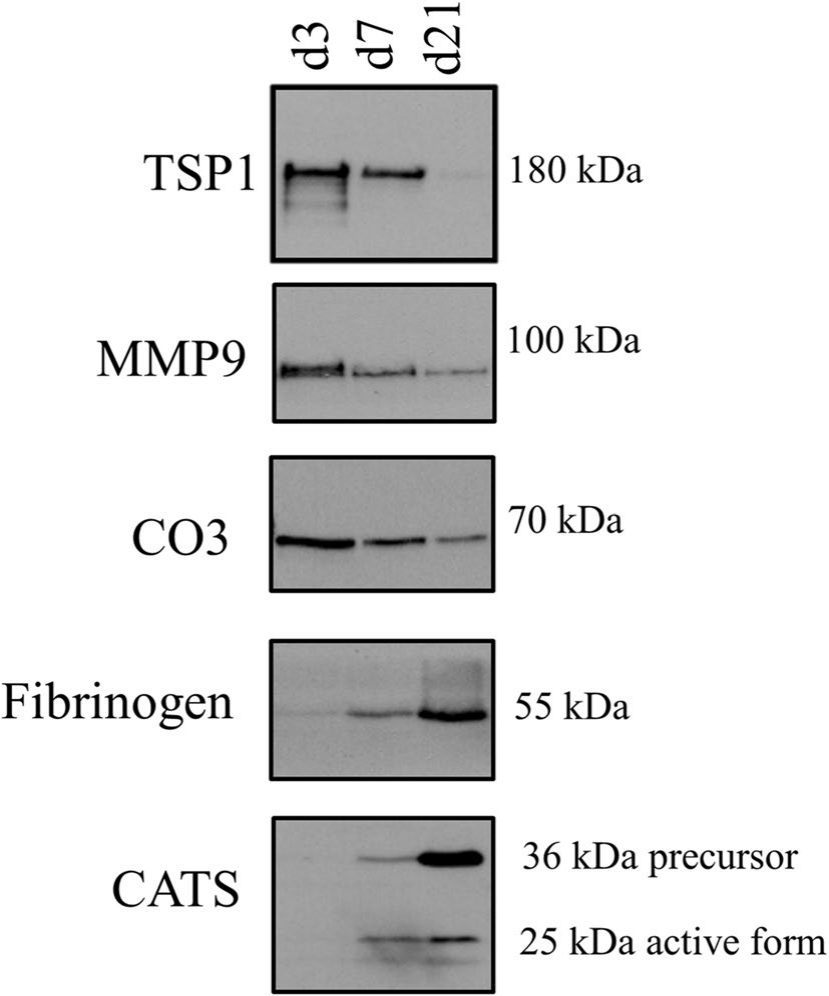
Western blot analysis confirms that TSP1, CO3 and MMP9 are up-regulated at day 3 and Fibrinogen and CATS are down-regulated at this time point. Validation analysis was performed in an independent cohort of L-PRF secretome samples. A representative image from 3 donors is shown.

Western blot analysis showed an enrichment in MMP9, TSP1 and CO3 at day 3 and a decrease in the amount of these proteins over time. On the contrary, fibrinogen and CATS showed increased levels over time, being the maximum at day 21. The results obtained are in line with the previous proteomic data obtained by SWATH (Fig. 3).

## Discussion

Different groups have measured specific growth factors released by PRC, compared their enrichment in different types of PRC and measured their kinetics over time^18,19,22,23^. Nevertheless, the total secretome released by PRC has not been yet analysed in detail. Recent advances in the proteomic field have allowed starting the analysis of PRC secretomes. Some studies have used different approaches to analyse the proteome of different PRC, although all of them were obtained using anticoagulants ^17,21^. Indeed, Yaprak et al. analysed the PRF secretome by 2D—LC/MS–MS finding a low number of identifications, only 35^20^.

In the present study, we performed the most detailed proteomic analysis of L-PRF secretome to date. Initially the secretome at day 3 was analysed by LC–MS/MS. Furthermore, growth factors at days 3 and 7 were quantified by ELISA and the differential protein releasate of L-PRF membranes at days 3, 7 and 21 of culture was analysed by SWATH.

The L-PRF secretome includes multiple proteins released by different blood cell types and with a variable dynamic range. Indeed, compared to typical PRC used in the clinical practice, the existence of leukocytes in this platelet rich concentrate membrane contributes to the presence of other proteins in the secretome, making it more complex. In this context, we found that fractionation of protein extracts by 1D-SDS-PAGE increased the proteome coverage. Thus, two complementary gel-based approaches were used to analyse the secretome at day 3, as indicated in the Methods section below. In the case of the differential secretome analysis, an initial profile based on differences by 1D-SDS-PAGE was followed by a more quantitative study based on SWATH.

A total number of 705 proteins were identified when profiling the L-PRF secretome at day 3. Clathrin mediated endocytosis, acute phase response and LXR/RXR activation, among others, were the top canonical pathways related to identifications at that day. Interestingly, Anitua et al.^17^ also found acute phase response and LXR/RXR between the top ten canonical pathways in the analysis of the proteins extracted with acetonitrile from their PRC. The presence of abundant proteins related to acute phase response such as fibrinogen, albumin, and complement cascade factors were also identified in the secretome and the lysates of other PRC^17,20,21^. In our study, clathrin mediated endocytosis is the most represented pathway mostly due to the presence of growth factors identifications such as PDGF and EGF in the L-PRF secretome. Interestingly, this type of endocytosis is an important route of internalization of tyrosin kinases receptors, which contribute to response to EGF^24^ and PDGF stimulating cell migration and proliferation^25^.

Other secretory pathways were found among the principal biological processes related to identifications at day 3. Indeed, the identification of ITA2B and CD9 in the secretome suggests the presence of platelet-derived EVs. On the other hand, proteins with antimicrobial activity such as MMP9, CAP7, PERM, BPI and CATG indicate neutrophil degranulation. These proteins released by neutrophils have not been described previously in PRC, possibly due to the fact that many of them do not contain leukocytes. L-PRF membranes contain platelets and leukocytes, although the proteomic analysis highlighted the presence of proteins derived from monocytes and CD4 lymphocytes in the secretome at day 3.

In order to know differences in the secretome of L-PRF over time, an initial 1D-SDS-PAGE analysis was performed at days 3 and 7, focusing on bands with different intensity between conditions. Following LC–MS/MS identification, more than 50% of proteins were found in both conditions; however, EGF was only identified at day 3. As growth factors are key for wound healing processes, a quantitative ELISA was performed in the same samples analysed by mass spectrometry as a complementary approach. According to this analysis, the majority of growth factors were found enriched at day 3, most probably due to a higher platelet and neutrophil degranulation at this time point. Only GDF15 was found down-regulated in all donors at day 3. Monocytes present in L-PRF differentiate to macrophages, which then release GDF15. At day 7 the number of macrophages probably would be higher than at day 3, for that reason the amount of this growth factor is increased at that day.

Some studies have found differences in the concentration and kinetic release of growth factors in different PRC^15,22,26,27^ even combined with xenografts^19^. Different methods used to prepare PRC are the responsible for the differences. In spite of the biological variability found in our study, we could get relevant data on the growth factor analysis between conditions that could be correlated with the proteomic data. Indeed, the most abundant growth factors identified by ELISA at day 3 (PDGFA, TGFB1 and EGF) were previously identified by proteomics at that day.

In this study, we wanted to go one-step further and quantify differences in the L-PRF secretome over time. For that purpose, new membranes from four different donors were collected and cultured for 21 days. Secretomes at day 3, 7 and 21 were analysed by a proteomic SWATH-based method. In this way, a total of 202 proteins were identified with significant differences. As expected, almost all proteins identified decreased over time, a possible explanation for that could be apoptosis of the different blood cells present in the L-PRF. MMP9, TSP1 and CO3 were among the proteins up-regulated at day 3; interestingly, these proteins were also found in the qualitative proteomic analysis. The upregulation of these proteins related to neutrophil and platelet-degranulation at day 3 was confirmed by western blotting. At the same time, in line with the theory exposed above, overexpression of some proteins over time such as CATD, CAH1 and PRDX2 could be explained by apoptosis. Indeed, two of them, CAH1 and PRDX2, are enzymes involved in detoxifying ROS and their higher presence over time indicate high levels of ROS and cellular stress.

CATD is a protein released by azurophilic granules of neutrophils. This protease was suggested as an inductor of apoptosis in mature neutrophils through caspase 8 activation and its release was delayed in absence of ROS^28^. In line with Conus results, the overexpression of CATD over time in L-PRF membrane indicate neutrophil apoptosis at days 7 and 21.

Surprisingly, fibrinogen was another protein whose levels increased over time. In the coagulation cascade, prothrombin turns into thrombin by the action of coagulation factors V and X. Afterwards, thrombin converts fibrinogen into fibrin, which is stabilized by factor XIII^29^. Platelet alpha granules release fibrinogen when platelets are activated. In line with the above, our results suggest platelet and neutrophil degranulation over time in the L-PRF membranes, which might be related to cell apoptosis. Absence of thrombin in the L-PRF secretome and the decreasing levels of prothrombin and coagulation factor V over time do not allow released fibrinogen to turn into fibrin, so fibrinogen accumulates. The results obtained by western blot measuring fibrinogen levels in an independent cohort of donors and membranes corroborate that fibrinogen accumulates over time in the L-PRF secretome. These higher levels of fibrinogen at days 7 and 21 might contribute to the wound healing properties of L-PRF membranes. The latter is in agreement with data from Rybarczyk and colleagues^30^ who demonstrated that Fibrinogen enhances both wound closure and cell proliferation to significantly shorten the time of wound closure in a dermal fibroblast model of tissue injury^30^.

Interestingly, another protein with increased levels in the secretome over time is CATS. A recent study has demonstrated its role to hydrolyze the α and β chains of fibrin. Indeed, it was suggested the possibility of multiple sites of cleavage along fibrin because CATS can even cleave fragments produced after plasmin-mediated fibrinolysis^31^.

The fibrinolytic properties of CATS, associated with its overexpression in the L-PRF secretome at later days, could be an explanation for the L-PRF membrane degradation over time. In addition, our validation experiments corroborated the overexpression of CATS in an independent cohort of donors at day 21. This supports the theory by Douglas et al.^31^ that suggests cathepsins are involved in the degradation of a fibrin network with endothelial cells and fibroblast encapsulated, which collapsed in an unpredictable an uncontrolled manner in days to weeks^32^. In addition to their fibrinolytic properties, CATS also participates in healing processes. Recently, Memmert et al.^33^ have studied the wound healing properties of CATS providing evidence that this enzyme stimulates periodontal ligament cells proliferation and migration^33^**.**

## Conclusion

To conclude, we analysed for the first time the secretome of L-PRF at day 3 and quantified differences in the secretome over time. The secretome profile at day 3 and the growth factors analysis performed at days 3 and 7 showed that EGF, PDGFA, TGFB1 and proteins related to platelet and neutrophil degranulation might be the responsible for the good wound healing results obtained after L-PRF application. Furthermore, differences found over time, including up-regulation of MMP9, TSP1 and CO3 and down-regulation of fibrinogen and CATS at day 3, show the reactions that are taking place in the biomembrane at each moment and contribute to understand the L-PRF biological properties.

## Methods

**Figure 4:**
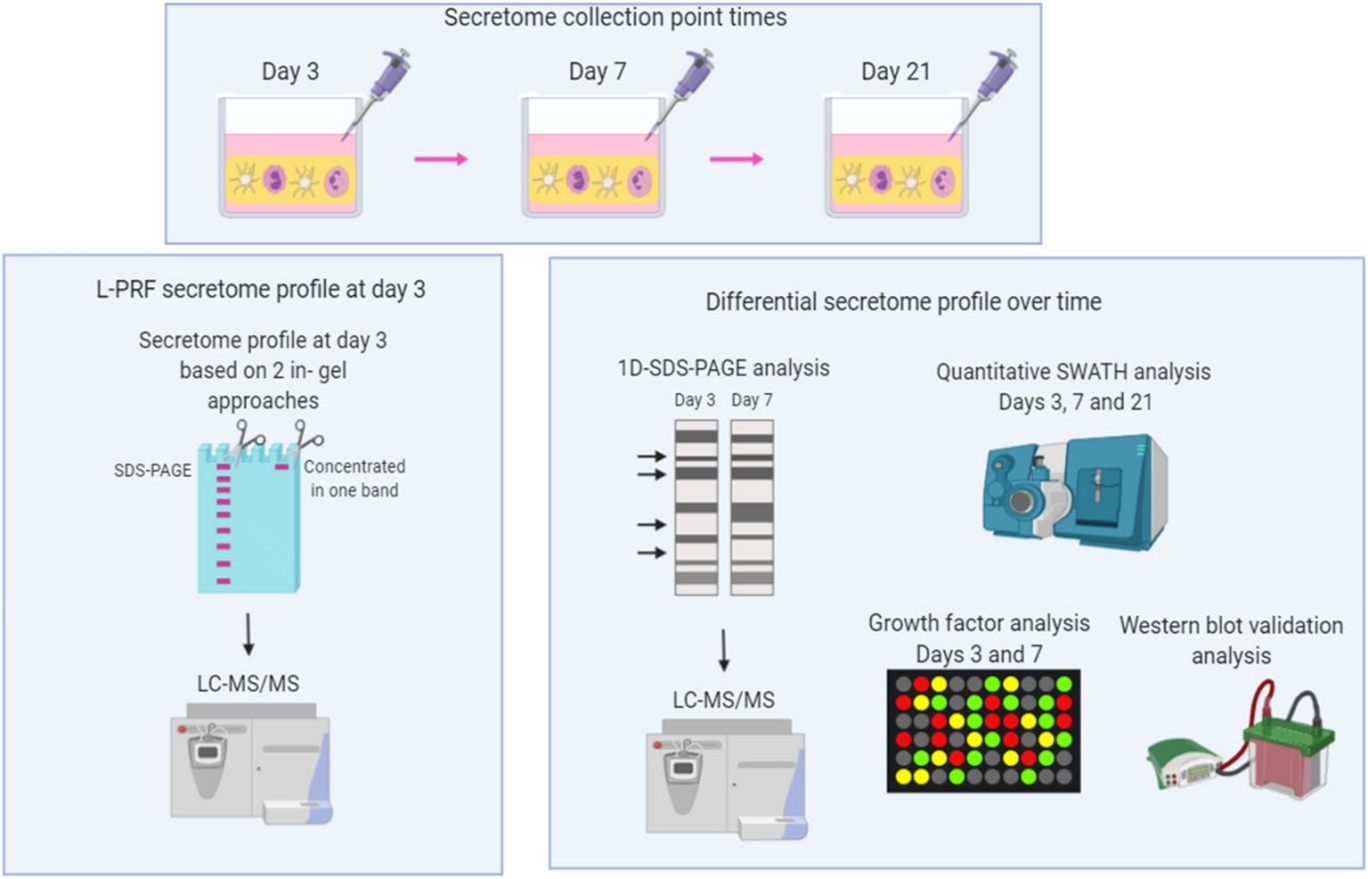
Experimental workflow of the study. Schematic representation of the methodology applied in this study. Created with Biorender.com.

The workflow of the experimental approach is shown in Fig. 4.

### L-PRF membranes obtention

This study was performed following the principles of the Declaration of Helsinki. The experimental protocol is part of a clinical assay approved by the Spanish Agency of Medicines and Medical Devices, which also covers ethical approval (EudraCT No. 2017-001068-39). Human venous whole blood from 11 healthy volunteers, 7 men and 4 women was collected into 9 ml glass-coated plastic tubes without anticoagulant (Intra-Spin, Intra-Lock Iberia, Madrid, Spain). Volunteers did not take any drug affecting blood coagulation or platelet aggregation for at least 10 days prior to blood sample collection. Informed consent was obtained from all subjects.

After blood extraction, the tubes were immediately centrifuged at 400 g for 12 min in an Intra-spin centrifuge (Intra-Lock Iberia, Madrid, Spain) in order to obtain the L-PRF clots. Clots were placed in a metal box and after 5 min of gravity pressure, L-PRF membranes were obtained.

### L-PRF culture and secretome collection

L-PRF membranes were placed into six-well plates and covered with 5 ml of DMEM medium (D5796 Sigma-Aldrich, St Louis, Missouri, USA) supplemented with 1% penicillin and streptomycin. Membranes were washed after 2 and 24 h with DMEM in order to eliminate the majority of plasma proteins and were cultured in fresh medium. Secretomes were collected at different time points after the last wash: day 3 (which represents the secretome released between 24 h and day 3), day 7 (which belongs to the secretome released between day 3 and 7) and day 21 (which belongs to the secretome released between day 7 and 21).

After collection, secretomes were centrifuged for 3 min at 3,000 g in order to discard debris contaminants. Secretomes were concentrated from 5 ml to 500 µl using Amicon Ultra-15 Centrifugal Filter (Merk, Millipore, Massachusetts, USA) of 3 kDa of size pore, and stored at -80ºC. Sample distribution per analysis is shown in Table 3.

**Table 3:** Sample distribution per analysis.

Samples	11 samples from healthy volunteers (biological replicates)				
Number of volunteers per study	4 (2 men, 2 women; median age, 29.5 years; range age, 23–67 years)			4 (2 men, 2 women; median age, 32 years; range age, 25–60 years)	3 (3 men; median age, 58 years; range age, 27–66 years)
Type of sample and approach performed	Pool for secretome profile	Pool for differential gel-based secretome profile	Individual samples for growth factor quantitative analysis	Pool for SWATH analysis	Individual samples for western blot validations

### Protein preparation and proteomic analysis

#### Protein precipitation and quantification

Samples used for proteomic analysis were precipitated in 20% trichloroacetic acid in acetone, as previously described^34^, and finally resuspended in a SDS buffer (2% SDS, 500 mM Tris pH 7.6 and 0.05 M Dithiothreitol).

Protein quantitation was done with Pierce 660 nm Protein Assay mixed with Ionic Detergent Compatibility Reagent following the manufacturer´s instructions (Thermo Fisher Scientific, Asheville, NC, USA).

#### Qualitative L-PRF secretome profile at day 3 of culture

Two approaches were performed in order to describe the L-PRF secretome profile at day 3. For the first approach, proteins from a pool of four donors were separated by 4–12% SDS-PAGE. After running, the gel was fixed (10% ethanol and 7% acetic acid) for one hour and stained overnight with Sypro Ruby (Thermo Fisher Scientific, Asheville, NC, USA). The gel was divided in 15 bands that were excised, and digested with trypsin, followed by LC–MS/MS analysis.

A second approach was based on loading the protein on a 11% SDS-PAGE gel just to concentrate the protein sample in a gel band that was excised, and proteins were in-gel digested with trypsin.

#### Differential proteomic profile between secretomes at days 3 and 7

Secretomes from membranes obtained from four donors were pooled in equal amounts at days 3 and 7. An initial proteome screening at days three and seven was performed by 1D-SDS-PAGE. Proteins were separated by 11% SDS-PAGE, loading 50 µg of each protein pool per lane. After electrophoresis, the gel was fixed (10% ethanol and 7% acetic acid) for one hour and stained overnight with Sypro Ruby (Thermo Fisher Scientific, Asheville, NC, USA). A total of eight protein bands (four per condition) corresponding to the differential profile were cut, digested with trypsin, and analysed by LC–MS/MS.

#### LC–MS/MS identification in the secretome profile analysis

After in-gel tryptic digestion of bands, peptides were extracted following an established protocol^35^, carrying out three incubations of 20 min each with 60% acetonitrile and 0.5% HCOOH. The resulting peptide extracts were pooled, concentrated and stored at − 20 °C.

Identifications were done using a Data Dependent Acquisition workflow (DDA) performed in a TripleTOF 6600 System (Sciex, Redwood City, CA, USA) following an established procedure^35^. Peptides were separated by Reverse Phase Chromatography using a micro liquid chromatography system (Eksigent Technologies nanoLC 400, Sciex, Redwood City, CA, USA) coupled to high-speed Triple TOF 6600 mass spectrometer (Sciex, Redwood City, CA, USA). Four microliters of sample were injected in the trap column YMCTRIART C18 (YMC Technologies, Teknokroma Analítica, Barcelona, Spain) with a 3 nm particle size and 120 Å pore size, switched on-line with the analytical silica-based reversed phase column YMC-TRIART C18 150 × 0.30 mm, 3 nm particle size and 120 Å pore size (YMC Technologies, Teknokroma Analítica, Barcelona, Spain). The loading pump delivered a solution of 0.1% formic acid in water at 10 μl/min. The micro-pump generated a flow-rate of 5 μl/min and was operated under gradient elution conditions, using as mobile phase A: 0.1% formic acid in water and as mobile phase B: 0.1% formic acid in acetonitrile. Peptides separation was done in a 90 min gradient ranging from 2 to 90% mobile phase B (mobile phase A: 0.1% formic acid, 2% acetonitrile; mobile phase B: 0.1% formic acid in 100% acetonitrile).

Data dependent Acquisition workflow was performed in a TripleTOF 6600 System (Sciex, Redwood City, CA, USA) using parameters previously set up^35^. Source and interface conditions were the following: ionspray voltage floating (ISVF) 5,500 V, curtain gas (CUR) 25, collision energy (CE) 10 and ion source gas 1 (GS1) 25. Instrument was operated with Analyst TF 1.7.1 Software (Sciex, Redwood City, CA, USA). Switching criteria was set to (m/z) 350–1,400 with charge state of 2–5, mass tolerance 250 ppm and an abundance threshold of more than 200 counts (cps). Former target ions were excluded for 15 s.

Peptide and protein identifications were against Human specific Uniprot database 2018_01 using Protein Pilot Software (version 5.0.1, Sciex, Redwood City, CA, USA) specifying the following parameters: iodoacetamide as variable and metionin oxidation as a fixed modifications. The false discovery rate (FDR) was set to 1% for both peptides and proteins.

#### Differential secretome protein quantitation at days 3, 7 and 21 by SWATH (sequential window acquisition of all theoretical mass spectra)

Protein samples from four new donors were pooled on equal amounts at days 3, 7 and 21. A total of 50 µg per pool were loaded on a 11% SDS-PAGE gel to concentrate the protein in a gel band that was excised and digested with trypsin.

Prior to proteomic analysis by SWATH, a MS/MS spectral library was constructed analyzing peptides by a shotgun data-dependent acquisition (DDA) approach by micro-LC–MS/MS. Samples from each condition (day 3, 7 and 21) were pooled using equal mixtures from the original ones in order to get a good representation of the peptides and proteins present in all samples. Run specifications were followed by an establish protocol^35^. Four microliters of each pool were separated into a micro-LC system Ekspert nLC425 (Eksigent Technologies, Dublin, CA, USA) using the same conditions as mentioned above. In this analysis, the gradient was 5% to 95% B for 30 min, 5 min at 90% B and finally 5 min at 5% B for column equilibration, for a total run time of 40 min. Peptides were directly injected into a hybrid quadrupole-TOF mass spectrometer Triple TOF 6600 (Sciex, Redwood City, CA, USA) operated with a data-dependent acquisition system in positive ion mode after elution. A Micro source (Sciex, Redwood City, CA, USA) was used for the interface between microLC and MS, with an application of 2,600 V. The acquisition mode consisted of a 250 ms survey MS scan from 400 to 1,250 m/z followed by an MS/MS scan from 100 to 1,500 m/z (25 ms acquisition time) of the top 65 precursor ions from the survey scan, for a total cycle time of 2.8 s. The fragmented precursors were then added to a dynamic exclusion list for 15 s; any singly charged ions were excluded from the MS/MS analysis.

Peptide and protein identifications were performed with Protein Pilot Software (version 5.0.1, Sciex, Redwood City, CA, USA), as described previously. MS/MS spectra of the identified peptides were used to generate the spectral library for SWATH peak extraction using the add-in for PeakView Software (version 2.2, Sciex, Redwood City, CA, USA) with SWATH Acquisition MicroApp (version 2.0, Sciex, Redwood City, CA, USA). Peptides with a confidence score above 99% (as obtained from Protein Pilot database search) were included in the spectral library.

Samples were analysed by SWATH-MS acquisition method^35^ performed in a TripleTOF 6600 LC–MS/MS system (AB Sciex, Redwood City, CA, USA) making 3 technical replicates per sample. For each sample set, the width of the 100 variable windows was optimized according to the ion density found in the DDA runs using a SWATH variable window calculator worksheet from Sciex (Sciex, Redwood City, CA, USA).

The targeted data extraction of the fragment ion chromatogram traces from the SWATH runs was performed by PeakView (version 2.2) using the SWATH Acquisition MicroApp (version 2.0). PeakView computed an FDR and a score for each assigned peptide according to the chromatographic and spectra components; only peptides with an FDR below 5% were used for protein quantitation. Protein quantitation was calculated by adding the peak areas of the corresponding peptides. The integrated peak areas (from PeakView) were directly exported to the MarkerView Software (AB Sciex, Redwood City, CA, USA) for relative quantitative analysis following Student´s t-test.

The mass spectrometry proteomics data have been deposited at the ProteomeXchange Consortium via the PRIDE^36^ partner repository with the dataset identifier PXD017963.

Username: reviewer76765@ebi.ac.uk; password: VgmYMhJu.

#### Systems biology analysis

Data were analysed through the use of IPA^37^ (QIAGEN Inc., https://www.qiagenbioinformatics.com/products/ingenuitypathway-analysis), String Software^38^, FunRich^39^ (https://www.funrich.org/) and Reactome^40^ (https://reactome.org/).

#### Quantitative growth factor array analysis

Quantibody Human Growth Factor Array (Raybiotech, Peachtree Corners, GA, USA) analysis was performed with the secretome samples collected at day 3 and 7 from other membranes from the same donors than in point 5.3.3. Secretomes from individual membranes and day conditions were concentrated by Amicon filters (Merk, Millipore, Massachusetts, USA) as described above.

After secretome concentration, protein concentration was determined with the Coomassie plus reagent (Thermo Fisher Scientific, Asheville, NC, USA). Array hibridation was done following the manufacturer instructions with a concentration of 500 µg/ml per sample (previously tested).

Array Scanning was performed by the manufacturer service and data was analysed using Quantibody Q-Analyzer Software version.8.40.4 (Raybiotech Peachtree Corners, GA, USA).

#### Western blot

Western blot was performed following an established protocol^41^ in an independent cohort of concentrated secretome samples collected from three donors at days 3, 7 and 21, to validate the results obtained by the proteomic SWATH-based approach. Before immunoblotting, proteins were separated in 11% SDS-PAGE gels; 10 µg of protein was loaded per lane.

The primary antibodies used were: rabbit anti-MMP9 antibody (3,852, Cell Signaling Technology, Danvers, Massachusetts, USA) dilution 1/1,000; mouse anti-TSP1 antibody (sc-73158, Santa Cruz Biotechnology, Dallas, Texas, USA) dilution 1/300; mouse anti-Fibrinogen antibody (sc-69775, Santa Cruz Biotechnology, Dallas, Texas, USA) dilution 1/1,000; mouse anti-CO3 antibody (sc-28294, Santa Cruz Biotechnology, Dallas, Texas, USA) dilution 1/500; and mouse-CATS antibody (sc-271619, Santa Cruz Biotechnology, Dallas, Texas, USA) dilution 1/500.

## Supplementary information

Supplementary Information.

Supplementary Table 1.

Supplementary Table 2.

Supplementary Table 3.
