# Supplementary material for: Deciphering the secretome of leukocyte-platelet rich fibrin: towards a better understanding of its wound healing properties
Source: Sci Rep. 2020 Sep 3;10:14571. doi: 10.1038/s41598-020-71419-7 (PMC7471699; doi:10.1038/s41598-020-71419-7)
Supplement: Supplementary file 1 — Supplementary Information. [file 41598_2020_71419_MOESM1_ESM.pdf]

Deciphering the secretome of leukocyte-platelet rich fibrin: towards a better understanding of its wound healing properties

Lidia Hermida-Nogueira[1], María N. Barrachina[1], Luis A. Morán[1], Susana Bravo[2], Pedro Diz[3], Ángel García[1#*], Juan Blanco[4#].

1 Platelet Proteomics Group, Center for Research in Molecular Medicine and Chronic Diseases (CIMUS), Universidade de Santiago de Compostela, and Instituto de Investigación Sanitaria (IDIS), Santiago de Compostela, Spain;

2 Proteomics Unit, Instituto de Investigación Sanitaria de Santiago de Compostela (IDIS), Santiago de Compostela, Spain.

3 Medical-Surgical Dentistry Research Group (OMEQUI), Faculty of Medicine and Odontology, Universidade de Santiago de Compostela (USC), and Instituto de Investigación Sanitaria de Santiago de Compostela (IDIS), Santiago de Compostela, Spain.

4 Periodontology Unit, and Medical-Surgical Dentistry Research Group (OMEQUI), Faculty of Medicine and Odontology, Universidade de Santiago de Compostela (USC), and Instituto de Investigación Sanitaria de Santiago de Compostela (IDIS), Santiago de Compostela, Spain.

Supplementary Figure 1

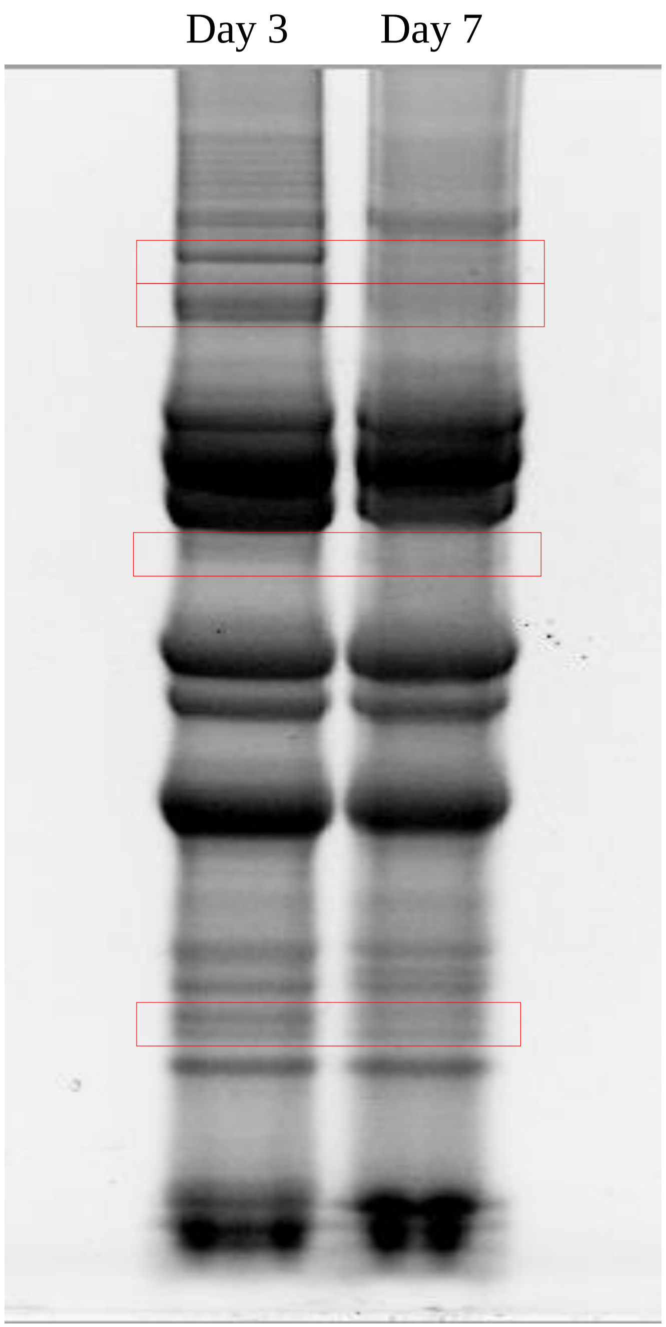

Supplementary figure 1. 1D-SDS-PAGE analysis of L-PRF secretomes at days 3 and 7. Four staining bands (marked in red box) showed clearly differences in intensity among conditions. Selected bands were excised, and proteins in-gel digested with trypsin and analysed by LC-MS/MS.

Supplementary Figure 2

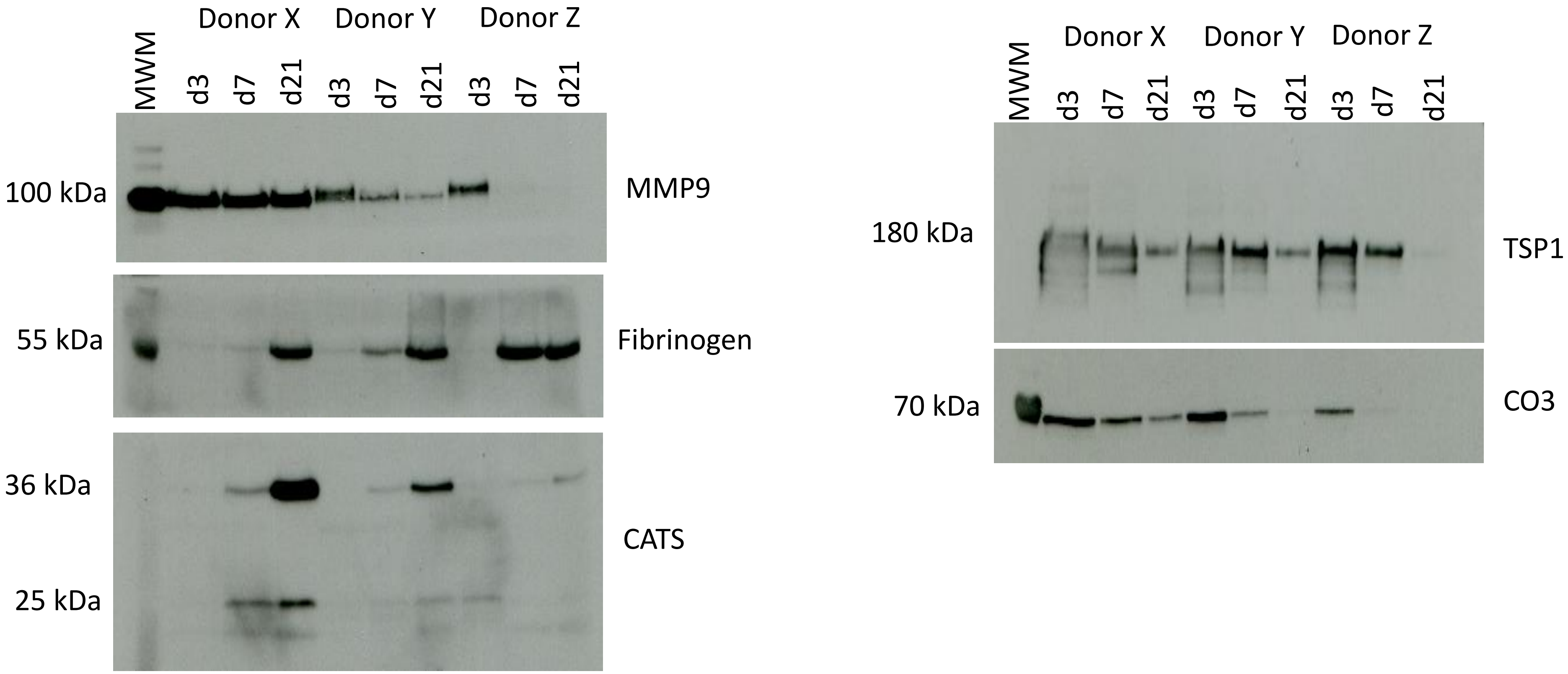

Supplementary figure 2. Western blot analysis of MMP9, Fibrinogen, CATS, TSP1 and CO3 in an independent cohort of L-PRF secretome samples at days 3, 7 and 21 (d3,d7,d21). Images show samples distributed in one gel and with the same time of exposure for the three donor samples displayed in each blot image. When necessary, alternative exposure times were obtained for a better analysis. MWM: molecular weight marker.
